# Metagenomic insights into the roles of *Proteobacteria* in the gastrointestinal microbiomes of healthy dogs and cats

**DOI:** 10.1002/mbo3.677

**Published:** 2018-06-17

**Authors:** Christina D. Moon, Wayne Young, Paul H. Maclean, Adrian L. Cookson, Emma N. Bermingham

**Affiliations:** ^1^ AgResearch Grasslands Research Centre Palmerston North New Zealand; ^2^ Riddet Institute Massey University Palmerston North New Zealand; ^3^ High‐Value Nutrition National Science Challenge Auckland New Zealand; ^4^ AgResearch Lincoln Research Centre Lincoln New Zealand; ^5^ AgResearch Hopkirk Research Institute Palmerston North New Zealand

**Keywords:** 16S rRNA gene, canine, fecal microbiome, feline, metagenome, *Proteobacteria*

## Abstract

Interests in the impact of the gastrointestinal microbiota on health and wellbeing have extended from humans to that of companion animals. While relatively fewer studies to date have examined canine and feline gut microbiomes, analysis of the metagenomic DNA from fecal communities using next‐generation sequencing technologies have provided insights into the microbes that are present, their function, and potential to contribute to overall host nutrition and health. As carnivores, healthy dogs and cats possess fecal microbiomes that reflect the generally higher concentrations of protein and fat in their diets, relative to omnivores and herbivores. The phyla *Firmicutes* and *Bacteroidetes* are highly abundant, and *Fusobacteria, Actinobacteria,* and *Proteobacteria* also feature prominently. *Proteobacteria* is the most diverse bacterial phylum and commonly features in the fecal microbiota of healthy dogs and cats, although its reputation is often sullied as its members include a number of well‐known opportunistic pathogens, such as *Escherichia coli, Salmonella,* and *Campylobacter*, which may impact the health of the host and its owner. Furthermore, in other host species, high abundances of *Proteobacteria* have been associated with dysbiosis in hosts with metabolic or inflammatory disorders. In this review, we seek to gain further insight into the prevalence and roles of the *Proteobacteria* within the gastrointestinal microbiomes of healthy dogs and cats. We draw upon the growing number of metagenomic DNA sequence‐based studies which now allow us take a culture‐independent approach to examine the functions that this more minor, yet important, group contribute to normal microbiome function.

## Abstract 2

The fecal microbiomes of healthy dogs and cats often include *Proteobacteria* at varying abundances. This phylum can have a sullied reputation as it contains a number of well‐known pathogenic members. We explored the functions of the *Proteobacteria* in fecal shotgun metagenome datasets from healthy dogs and cats. The *Proteobacteria* appeared to be enriched for functions that are consistent with a role in helping to maintain the anaerobic environment of the gut for normal microbiome function.

## 1 INTRODUCTION

Domesticated dogs and cats are popular companion animals, and interest in the importance and impact of their gastrointestinal (GI) microbiota on their health and wellbeing is a growing area (Deng & Swanson, 2015). The GI microbiota is a dense and diverse group of microorganisms that reside in the GI tracts of their hosts, and ferment available substrates derived from both diet and the host. In addition to their more prominent role in digestion, they also provide vitamins and substrates that are required by the host (Leblanc et al., 2013), provide specific energy sources required for intestinal epithelium integrity and contribute to its normal function (Louis & Flint, 2009), modulate the immune system (Kelly et al., 2004), and can protect the GI tract from colonization by pathogens (Ng et al., 2013). Moreover, the contributions of the GI microbiota to signaling between the central and enteric nervous system (the gut–brain axis) are becoming better understood (Perry et al., 2016), and impact on brain development and behavior.

In mammals, both major dietary shifts and host phylogeny have played influential roles in shaping the composition of gut microbiota over evolutionary timescales (Groussin et al., 2017). While modern domestic dogs and cats are generally fed a variety of commercially manufactured pet foods, consistent with the predatory lifestyles of their carnivorous ancestors, their metabolism and digestive system anatomies are adapted to diets that are rich in animal proteins and fat. Carnivore GI tracts are relatively shorter than those of omnivores and herbivores, reflecting the lower retention times required for the digestion of meat. The GI tract walls are typically much thicker to withstand bone fragments in the diet (Bosch, Hagen‐Plantinga, & Hendriks, 2015). Domestic cats are classified as obligate carnivores. However, over the course of domestication, dogs appear to have adapted to eating small amounts of starch and vegetation (Axelsson et al., 2013), thus, are largely considered facultative carnivores (Swanson et al., 2011). Modern domestic dogs and cats are fed diets that vary considerably in both format and nutrient profile. Commercially manufactured diets are typically produced in canned or kibbled formats, and in the case of kibbled diets, often contain considerable amounts of plant‐based carbohydrate, although it is recognized that neither dog nor cat has a nutritional requirement for carbohydrate (AAFCO, 2016). Raw meat‐based diets are also increasing in popularity due to purported benefits associated with their apparent biological fit with nutritional requirements, such as higher macronutrient digestibility (Bermingham, Maclean, Thomas, Cave, & Young, 2017). However, such diets have also come under criticism due to various concerns that include an inherent risk of bacterial and parasite contamination of the diet (van Bree et al., 2018), and shedding of infectious agents to humans, particularly those of high risk such as the elderly, immunocompromised, young, and pregnant (Freeman, Chandler, Hamper, & Weeth, 2013).

## 2 CHARACTERIZATION OF DOG AND CAT GI MICROBIOTA

Our understanding of the impact of diet on the GI microbiota, and their subsequent impact on host health and wellbeing, has grown in the last decade with the application of high‐throughput sequencing technologies. Traditionally, characterization of the dog and cat GI microbiota has been based on culture‐based approaches, where culture‐based insights into the diversity of microbes of dogs and cats to date have been fairly limited beyond pathogenic agents (Johnston et al., 2001). However, recent developments in systematic culture‐based methodologies have resulted in considerable progress cultivating the previously ‘uncultivable’ species of the human gut microbiota (Lagkouvardos, Overmann, & Clavel, 2017). Similar efforts for the dog and cat GI microbiota are yet to be undertaken.

**Figure 1:**
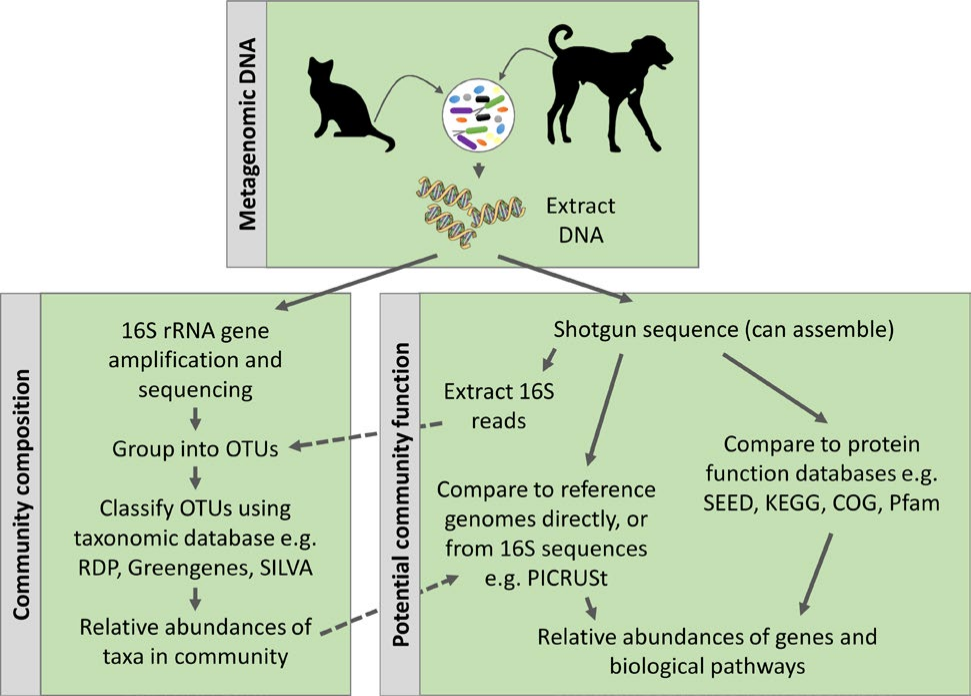
Common metagenomic DNA‐based analyses to define the community composition and function of the microbial communities in the dog and cat GI tract. Metagenomic DNA is extracted from a GI content sample, usually freshly voided fecal material, which contains microbial community members. The microbial community composition (see lower left box) is most commonly determined by amplifying variable regions of the 16S rRNA gene and sequencing the resulting amplicons. Similar 16S rRNA sequences are grouped into Operational Taxonomic Units (OTUs), which can be compared to specialized 16S rRNA sequence‐based taxonomic databases (e.g., RDP, Greengenes, SILVA) to assign taxonomic identities. The community can be described in terms of the relative abundance of the taxa present, and/or their phylogenetic relationships. To enable the potential function of the microbial community to be explored (see lower right box), metagenomic DNA is directly shotgun sequenced. The functional potential of the community can be determined by comparing the sequences to reference genomes, gene catalogs, or functional databases (e.g., SEED, KEGG, and COG). This allows the community to be described in terms of the relative abundances of its genes and pathways. More recently, inferences of community function may be made from 16S rRNA‐based taxonomic profiles using reference genome information implemented in software such as PICRUSt. Moreover, community composition may be deduced from shotgun sequenced DNA by capturing the 16S rRNA gene sequence reads, with classification using dedicated 16S rRNA databases

Our understanding of GI microbiome composition and function in healthy dogs and cats has been vastly enhanced by culture‐independent studies facilitated by molecular approaches, in particular, those enabled by advances in high‐throughput sequencing (Figure 1). The GI microbiomes of companion animals are commonly explored through fecal samples, which are far less invasive to obtain than *in situ* GI tract content samples. Total DNA extracted from these samples (metagenomic DNA) may be amplified using PCR, commonly of informative marker genes such as the 16S ribosomal RNA (rRNA) genes, and sequenced. These data can provide detailed information on the taxonomic composition of the microbiota (Sogin et al., 2006). Alternatively, metagenomic DNA may be sequenced directly to gain information on both microbiota function, and taxonomic composition, which may be extracted from 16S rRNA or other informative marker genes within the shotgun dataset (Deusch et al., 2015; Swanson et al., 2011; Tun et al., 2012; Young, Moon, Thomas, Cave, & Bermingham, 2016). The fecal microbiota of dogs and cats contain all three domains of life – Archaea, Bacteria, and Eukarya. The Bacteria comprise the vast majority of the community, with recent metagenome‐based estimates indicating that they make up ~98% (Swanson et al., 2011; Tun et al., 2012). Estimates for the minor components of the community, Archaea, Eukarya, and viruses, ranged from ~0.2% to 1.1%, ~0.4% to 1.2%, and ~0.1% to 0.3%, respectively (Deusch et al., 2014; Swanson et al., 2011; Tun et al., 2012). Microbiota composition also varies along the GI tract, where the hindgut compartments contain the highest microbial diversity in comparison to the stomach and small intestine (Honneffer, Steiner, Lidbury, & Suchodolski, 2017; Ritchie, Steiner, & Suchodolski, 2008; Suchodolski, Camacho, & Steiner, 2008). This observation is consistent with the role of the colon and cecum as the main site of fermentation in monogastric mammals, and that the physiological conditions along the GI tract vary in their ability to support microbial life.

Bacterial 16S rRNA gene surveys have shown that, like other mammals, dogs and cats harbor complex GI microbial communities whose taxonomic compositions vary not only by diet but also factors such as age (Deusch et al., 2015), incidence of metabolic disorders (e.g., obesity, diabetes) (Bell et al., 2014; Handl et al., 2012; Park et al., 2015) and intestinal issues (e.g., IBD, diarrhea) (Guard et al., 2015; Suchodolski, Markel, et al., 2012; Suchodolski et al., 2015). In clinically healthy dogs and cats, *Firmicutes* and *Bacteroidetes* are generally the dominant phyla found in the fecal microbiome, with *Fusobacteria, Actinobacteria,* and *Proteobacteria* also featuring prominently (Deng & Swanson, 2015; Garcia‐Mazcorro & Minamoto, 2013). In the dog and cat, the general roles and functions of these bacterial phyla are assumed to be similar to their roles in the gut microbiota of model organisms such as humans and rodents, for which, more information is available. However, there is building evidence that this may not be a viable assumption. For example, *Fusobacterium* appears to be associated with inflammatory bowel disease (IBD) and colorectal cancer in humans, but not necessarily in dogs (Vázquez‐Baeza, Hyde, Suchodolski, & Knight, 2016), where they have been found in high abundance in healthy dogs fed a raw red meat compared to a kibble diet (Bermingham et al., 2017). *Fusobacterium* has also generally been found in higher concentrations in healthy carnivore hosts (Ley et al., 2008).

The *Proteobacteria* are commonly occurring in healthy mammalian GI microbiomes. *Proteobacteria* are more abundant in dogs and cats fed high protein diets, but are frequently highlighted as a microbial group of particular concern to veterinarians and pet owners as they include a number of clinically important gastrointestinal pathogens, such as diarrheagenic *Escherichia coli, Campylobacter jejuni, Klebsiella pneumoniae*,* Salmonella typhimurium,* and *Yersenia enterocolitica* (Kil & Swanson, 2011) that may affect the health and wellbeing of both the pet and its owner. Moreover, high abundances, or “blooms”, of *Proteobacteria* in the GI tract, have been suggested as a microbial signature of dysbiosis in humans and mice (Shin, Whon, & Bae, 2015). In dogs and cats, members of the *Proteobacteria* have significantly increased abundances in individuals with gut inflammation (Minamoto et al., 2015; Suchodolski, Dowd, Wilke, Steiner, & Jergens, 2012; Suchodolski, Markel, et al., 2012; Suchodolski et al.,^2015^; Vázquez‐Baeza et al., 2016) and metabolic disorders (Park et al., 2015). In addition, companion animals may act as reservoirs for antimicrobial resistant bacteria, further contributing to public health concerns (Rubin & Pitout, 2014).

## 3 PROTEOBACTERIA

The *Proteobacteria* are named after Proteus, a Greek god of the sea, capable of assuming many forms in reflection of the broad morphological and metabolic diversity contained within this phylum (Stackebrandt, Murray, & Trüper, 1988). *Proteobacteria* is the largest bacterial phylum, and six classes and over 116 families are currently recognized (http://www.bacterio.net/). Members are Gram negative, and play a variety of roles in a range of diverse microbial ecosystems, such as in aquatic, soil, plant, and animal niches. Within anaerobic gastrointestinal environments, the *Gammaproteobacteria* are often the most prevalent class of *Proteobacteria* present. Unlike the majority of microbes in the GI microbiome that are strict anaerobes, *Proteobacteria* are often facultatively or obligately anaerobic, thus are able to tolerate a range of oxic conditions. As such, it is postulated that the *Proteobacteria* contribute to homeostasis of the anaerobic environment of the GI tract, and hence, the stability of the strictly anaerobic microbiota. Moreover, in humans and other mammals, facultative anaerobes including *Proteobacteria* are among the earliest colonizers and dominant members in the neonatal gut, which is abundant in oxygen immediately post partum. By consuming oxygen, and lowering redox potential, it has been speculated that the *Proteobacteria* play a key role in preparing the gut for successive colonization by the strict anaerobes required for healthy gut function (Shin et al., 2015). *Proteobacteria* can also colonize the mucus layer of the acid secreting stomach, where several *Helicobacter* species have been identified and isolated from dogs and cats (Haesebrouck et al., 2009). Gastric *Helicobacter* isolates are able to increase the pH in their local external environment *via* the action of urease to liberate alkaline ammonium salts from urea (Sidebotham, Worku, Karim, Dhir, & Baron, 2003). Many clinically healthy dogs and cats are infected with *Helicobacter* species, although these species can also associate with inflammation of the gastric mucosa and have been hypothesized to cause gastric lymphoma in cats (Haesebrouck et al., 2009).

*Proteobacteria* are able to grow on a range of organic compounds including protein, carbohydrates, and lipids. Recent studies of the human gut microbiome have shown that while the *Firmicutes* and *Bacteroidetes* possess many conserved genes that contribute to the functional redundancy of the microbiome; despite their relatively lower abundance, the *Proteobacteria* contribute to much of the functional variation (Bradley & Pollard, 2017). This observation suggests that the major sources of taxonomic variation in microbiota do not necessarily contribute the most variation in function (Bradley & Pollard, 2017). As many observations regarding the *Proteobacteria* have been based on human or rodent models, whether these extend to their roles in the microbiomes of the domestic dog and cat requires further investigation. In this study, we review and further analyze the growing number of available community composition and metagenomic datasets based on the GI and fecal microbiota of healthy dogs and cats to better understand the prevalence, diversity, and roles of the *Proteobacteria* within these hosts.

## 4 ABUNDANCE OF PROTEOBACTERIAIN DOG AND CAT FECAL MICROBIOMES *VIA* 16S rRNA GENE ANALYSES

A summary of the prevalence and diversity of *Proteobacteria* in clinically healthy dog and cat fecal microbiome studies, based on the information reported in 16S rRNA gene survey studies for which key technical information was available, is shown in Tables 1 and 2. These studies have typically analyzed thousands to tens of thousands of bacterial sequences per sample, and compared to traditional culture and clone‐based approaches, have enabled more detailed insight into the *Proteobacteria* which typically comprise only a few percent of 16S rRNA gene sequences. Within these studies, variations in fecal microbial community profiles, even for cohorts of animals receiving the same diet, could be considerable, and likely impacted by the complex interplay between host genetics and physiology of the individual, with its environment and diet.

**Table 1:** Prevalence of *Proteobacteria* in healthy dog fecal microbiota studies based on 16S rRNA gene sequencing

Study description^a^	Subjects/age	Sequencing and analysis methods^b^	*Proteobacteria* abundance and general microbiome descriptions^c^	Reference
General microbiome characterization				
Fecal microbiome	Mixed age, breed, and sex (N = 12)	454, V1‐V3 region, NCBI	Median values for all *Proteobacteria* genera detected were 0%, and in general, upper end of range was <0.1%. Prevalent genera were *Anaerobiospirillum* (0%–1.88%), *Pseudomomas* (0%–0.14%), and *Succinivibrio* (0%–0.1%)	(Handl et al., 2011)
Fecal microbiome	Mixed age, breed, sex NS (N = 6)	454, V1‐V3 region, NCBI	*Proteobacteria* ranged from 0% to 17%. Highest abundance in dogs fed the Beneful (10%–17%) and Science Diet (~10%) diets, but negligible in other diets. *Alphaproteobacteria (Hyphomicrobiaceae)* predominated. *Proteobacteria* were observed in 4 of the 6 dogs, and was the third‐most abundant phylum after *Firmicutes* and *Actinobacteria*	(Garcia‐Mazcorro et al., 2012)
Fecal microbiome	Mixed age, miniature schnauzer, mixed sex (N = 11)	454, V1‐V3 region, RDP	Mean of 11.31% *Proteobacteria* observed across all dogs. Highest (21.6%) was from dogs fed a “Trial” diet. *Gammaproteobacteria* (*Succinivibrionaceae* and *Enterobacteriaceae*) were predominant. *Proteobacteria* were observed in all individuals and was generally the fourth‐most abundant phylum after *Fusobacteria, Bacteroidetes,* and *Firmicutes*	(Hand et al., 2013)
Diet, pre‐ and probiotic trials				
Dry control (30% CP, 19% fat, 1.4% fiber) vs. beet pulp containing diet (28% CP, 21% fat, 4.5% fiber)	~20 months, mongrel and hound crosses, female (N = 6)	454, V3 region, RDP	*Proteobacteria* ranged ~4%–10.5% (mean 7%), with no significant difference between diet treatments. *Proteobacteria* were observed in all six dogs, and was generally the fourth‐most abundant phylum after *Firmicutes*,* Bacteroidetes,* and *Fusobacteria*	(Middelbos et al., 2010)
Diet (various commercial) with synbiotic administration	Mixed age, breed, and sex (N = 12)	454, V1‐V3, NCBI	*Proteobacteria* was the fifth‐most abundant phylum after *Firmicutes*,* Bacteroidetes*,* Actinobacteria,* and *Fusobacteria*	(Garcia‐Mazcorro et al., 2011)
Dry control (31% CP, 14% fat, 3.0% fiber) vs. cooked navy bean diet (30% CP, 14% fat, 3.0% fiber)	Mixed age, breed, and sex (N = 10)	454, V4‐V6 region, NCBI	Diet did not have a significant effect on *Proteobacteria* abundance (0.79% in control, 1.68% in navy bean diet). The *Enterobacteriaceae* were the most abundant family of *Proteobacteria. Proteobacteria* was the fourth‐most abundant phylum after *Firmicutes, Actinobacteria,* and *Fusobacteria*	(Forster et al., 2012; Kerr et al., 2013)
Potato fiber at five concentrations in an extruded diet (25% CP, 13%–15% fat, 10.8%–11.4% fiber)	~6 years, hound, female (N = 10)	454, V4‐V6 region, Greengenes	*Proteobacteria* abundance increased with increasing potato fiber concentration (from 1.5% to 2.8%), although this increase was not significant. *Sutterella* and *Succinivibrio* were the most abundant *Proteobacteria* genera, and *Proteobacteria* were the third‐most abundant phyla after *Firmicutes* and *Fusobacteria*	(Panasevich et al., 2015)
Beef and chicken raw meat diets with and without 1.4% inulin or 1.4% yeast cell wall (25%–30% CP, 45%–50% fat)	~5.5 years, beagle, female (N = 6)	454, V4‐V6 region, NCBI	*Proteobacteria* ranged from 4.1% to 5.8% in abundance across diets. Beef diets increased the abundance of *Escherichia* and decreased the abundance of *Anaerobiospirillum* compared to chicken diets. Inulin reduced the abundance of *Enterobacteriaceae* and *Escherichia*. Overall, *Proteobacteria* were observed in all six dogs, and was generally the fourth‐most abundant phylum after *Fusobacteria, Firmicutes,* and *Bacteroidetes*.	(Beloshapka et al., 2013)
Raw meat (76% CP, 18% fat, 0.6% fiber) vs. kibble (30% CP, 27% fat, 2.4% fiber)	~5.8 years, harrier hound, mixed sex (N = 15)	Illumina MiSeq, V4‐V6, Greengenes 13_8	Dogs fed kibbled diet averaged 1.27% *Proteobacteria* (0.2%–4.7%), compared to 0.55% (0.08%–1.87%) when fed raw meat diet. *Proteobacteria* (mainly *Gammaproteobacteria*) were observed in all dogs, and was the fourth‐most abundant phylum after *Firmicutes*,* Bacteroidetes,* and *Fusobacteria*.	(Bermingham et al., 2017)
Diet (various) supplemented with 225 mg FOS+inulin prebiotic for 16 days	Mixed age, breed, and sex (N = 10)	454, V4‐V5 region, Greengenes, PICRUSt	*Sutterella* was detected among more dogs after administration of prebiotic.	(Garcia‐Mazcorro et al., 2017)
Diet (various) supplemented with FOS+inulin prebiotic at 0.5% of DMI	Mixed age, breed, and sex (N = 10)	454, V4‐V5 region, Greengenes, PICRUSt	Administration of prebiotic did not significantly impact the relative abundance of *Proteobacteria* subtaxa.	(Garcia‐Mazcorro et al., 2017)
Dry control (27% CP, 11% fat, 2.8% fiber) vs. raw meat based (26% CP, 18% fat, 0.7% fiber) diet	Mixed age, boxer, female (N = 8)	Illumina MiSeq, V3‐V4, RDP	Dogs fed the control diet had an average of 2.4% *Proteobacteria* compared to 4.4% when fed the raw meat‐based diet. *Sutterellaceae* (*Betaproteobacteria*) were prevalent among treatments and *Enterobacteriaceae* (*Gammaproteobacteria*) predominated in raw meat fed dogs. *Proteobacteria* was the fourth‐most abundant phylum after *Firmicutes*,* Bacteroidetes,* and *Fusobacteria*.	(Sandri et al., 2017)
Natural (90% raw meat plus vegetables) vs. commercial feed (18%–21% CP, 8%–10% fat)	Mixed age, small breeds, and sex (N = 11)	Illumina MiSeq, V3‐V4, EzBioCloud	*Proteobacteria* abundance averaged 0.86% in dogs fed the natural diet where it was the fifth‐most abundant phylum, as compared to 8.67% for the commercial diet‐fed dogs, where it was the third‐most abundant. Within the latter treatment, *Escherichia coli* comprised 7.3% of sequences.	(Kim et al., 2017)
Diet shifted from a commercial dry diet to increasing proportions of boiled minced beef added, then reversion to dry diet.	Mixed age, breed, and sex (N = 11)	Illumina MiSeq, V3‐V4, Greengenes	*Sutterella* and *Anaerobiospirillum* were the predominant genera present, and like *Proteobacteria* (~4%–6%), decreased in relative abundance as beef content was increased. *Proteobacteria* was the third‐most abundant phylum after *Firmicutes* and *Fusobacteria* in dogs on all treatments.	(Herstad et al., 2017)

Notes

a CP, crude protein; FOS, fructooligosaccharide.

b 454 refers to 454 GS FLX Titanium sequencing.

c If available, descriptions of sub‐phylum level abundance and *Proteobacteria* prevalence among individuals are given. The level of taxonomic detail provided in each study varied and taxonomy (OTU) tables were not always available.

**Table 2:** Prevalence of *Proteobacteria* in healthy cat fecal microbiota studies based on 16S rRNA gene sequencing

Study description^a^	Subjects/age	Sequencing and analysis methods^b^	*Proteobacteria* abundance and general microbiome descriptions^c^	Reference
General microbiome characterization				
Fecal microbiome	Mixed age, breed, and sex (N = 12)	454, V1‐V3 region, NCBI	Median values for all *Proteobacteria* genera were 0%, and the upper end of range for genus abundance was <0.1%, apart from *Succinivibrio,* which was 0.51%	(Handl et al., 2011)
Diet, pre‐ and probiotic trials				
Diet (various commercial) with synbiotic administration	Mixed age, breed, and sex (N = 12)	454, V1‐V3, NCBI	*Proteobacteria* was the fourth‐most abundant phylum after *Firmicutes*,* Actinobacteria,* and *Bacteroidetes*. Synbiotic did not appear to impact abundance of any class, order, family, or genus in these phyla	(Garcia‐Mazcorro et al., 2011)
Kitten fecal microbiome of kittens fed kibble (35% CP, 20% fat, 1.8% fiber) vs. canned (45% CP, 38% fat, 1.5% fiber), from mothers fed canned or kibbled diets (crossover design).	Kittens (8 and 17 weeks), shorthair, mixed sex (N = 20)	454, V1‐V3 region, RDP	Average *Proteobacteria* abundance was 1.2%–4.9% across treatments, with 4.9% in the canned‐fed kitten from kibble‐fed mother. *Sutterella* abundance significantly differed across treatments at 0.79% in canned‐fed kittens from canned‐fed mothers, compared to the three other treatments (0.24%–0.27%). *Proteobacteria* were observed in 19/20 kittens, and was the fourth‐most abundant phylum after *Firmicutes, Bacteroidetes, and Fusobacteria*	(Bermingham, Kittelmann, et al., 2013)
Dry format diet (33% CP, 11% fat, 1.9% fiber) vs. wet (42% CP, 42% fat, 1.6% fiber)	Mixed age, shorthair, mixed sex (N = 12)	454, V1‐V3 region, RDP	Average *Proteobacteria* abundance was 1.1% in the wet vs. 0.4% in the dry diet treatment, where *Anaerobiospirillum* and *Sutterella* were significantly more abundant (0.4% and 0.6%, respectively) in the wet treatment. *Proteobacteria* were detected in all animals, and was the fifth‐most abundant phylum after *Firimicutes, Bacteridetes/Fusobacteria,* and *Actinobacteria*	(Bermingham,Young et al., 2013)
MPMC diet (34% CP, 19% fat, 6.9% fiber) vs. HPLC diet (53% CP, 24% fat, 2% fiber)	Kittens (8, 12, and 16 weeks), domestic shorthair, mixed sex (N = 14)	454, V4‐V6 region, NCBI	*Proteobacteria* abundance, averaged per time point was 3.3%–3.7% in HPLC and 0.1%–1.1% in the MPMC groups	(Hooda et al., 2013)
Diet (various) supplemented with 225 mg FOS+inulin prebiotic for 16 days	Mixed age, breed, and sex (N = 10)	454, V4‐V5 region, Greengenes, PICRUSt	Linear discriminant analysis effect size identified *Gammaproteobacteria* abundances contribute bacterial community differences between pre‐ and during prebiotic administration. *Gammaproteobacteria* were detected in 9/10 cats prior to, but only 4 cats after, prebiotic administration suggesting that the prebiotic selected against this class	(Garcia‐Mazcorro et al., 2017)
Kibble (composition) vs. raw meat‐based diets (composition); with and without prebiotic.	Mixed age, shorthair, mixed sex (N = 12)	Illumina MiSeq, V3‐V4, Greengenes	*Proteobacteria* were less abundant in cats fed the raw meat diet (average 2.4% on kibble vs. 0.4% on raw meat), and were dominated by *Anaerobiospirillum* (0.6%–2.5%) and *Succinivibrio* (1.2%–2.1%) in the kibble diet‐fed cats. When fed the kibble diet *Proteobacteria* were the third‐most abundant phylum after *Firmicutes* and *Bacteroidetes*. When fed the raw meat diet, *Fusobacteria* was third‐most abundant and *Proteobacteria* was fourth	Butowski et al., unpublished

Notes

a CP, crude protein; FOS, fructooligosaccharide.

b 454 refers to 454 GS FLX Titanium sequencing.

c If available, descriptions of sub‐phylum level abundance and *Proteobacteria* prevalence among individuals are given. The level of taxonomic detail provided in each study varied and taxonomy (OTU) tables were not always available.

When comparing the data generated from different studies, care must be exercised as technical differences between the analytical methods employed will generate biases in the data reported. It is well recognized that variation in microbial community composition may arise from differences in factors such as sample storage method, DNA extraction procedure, PCR primer sequences and 16S rRNA gene region amplified, PCR amplification conditions, and sequencing technology (Boers, Jansen, & Hays, 2016). In particular, differences in cell lysis treatments for DNA extraction may bias the representation of *Proteobacteria*, with lower recovery of intact DNA from Gram‐negative bacteria associated with harsher lysis conditions (Yuan, Cohen, Ravel, Abdo, & Forney, 2012). The choice of bioinformatics procedures used (including sequence data quality filtering, chimera detection, and low‐abundance OTU cut‐offs) and, in particular, how taxonomic assignments are made, also bias the microbiota composition reported. A range of reference databases are commonly used (Cole et al., 2007; Desantis et al., 2006; Pruesse et al., 2007) that are frequently updated as microbial taxonomies are refined. Differences between these databases (Balvočiūtė & Huson, 2017) and updates over time may impact the degree and level of taxonomic detail that assignments have been made. Furthermore, differences between studies in the level of detail that data were reported to (e.g., from taxon frequencies per individual animal to group medians and ranges, and the level of taxonomic rank reported to) were very apparent, as well as the numbers of individuals examined, and these factors also limit the degree to which data can be directly compared between studies. An understanding of the range of microbiota compositions that are ‘normal’ for healthy individuals is only beginning to be established (AlShawaqfeh et al., 2017; Suchodolski, Dowd, et al., 2012). In general, *Proteobacteria* were detected from almost all individuals examined, which suggests they play a fairly essential role in the GI microbiomes of healthy pets.

### 4.1 Proteobacteria in dogs

Studies of the fecal microbiomes of clinically healthy adult dogs (Table 1) indicate that the relative abundances of *Proteobacteria* are generally higher and more variable than in cats (Table 2). In general, *Proteobacteria* comprised from 0% to 22% of 16S rRNA reads of the fecal microbiomes reported in dogs, although high abundances at the top of this range were seldom observed. In a study of six privately owned dogs of a variety of breeds and ages, the median *Proteobacteria* relative abundance observed was 1%, although ranged from 0% to 17% across the individuals (Garcia‐Mazcorro, Dowd, Poulsen, Steiner, & Suchodolski, 2012). In a research colony group of 11 adult miniature Schnauzers fed a range of commercial diets, the average abundance of *Proteobacteria* was 11.3%, with a range from 0.03% to 21.6%, and *Sutterella* comprised a large majority of the *Proteobacteria* detected (Hand, Wallis, Colyer, & Penn, 2013). In contrast to these two studies, low proportions of *Proteobacteria* were detected in a pyrosequencing‐based analysis of the fecal microbiota of 12 privately owned healthy dogs (Handl, Dowd, Garcia‐Mazcorro, Steiner, & Suchodolski, 2011). In this study, data were reported at the genus level, and all *Proteobacteria* genera had a median percent of sequences as 0%. The most abundant genus was *Anaerobiospirillum* (family *Succinivibrionaceae*), which occurred at 0% to 1.88%, although again, the median was 0%. Observations of the healthy subjects in epidemiological studies also show *Proteobacteria* at a range of abundances. For example, in a study that examined microbial dysbiosis in dogs with acute diarrhea, the control group of 13 healthy dogs displayed a median of 0.1% *Proteobacteria* (with range 0.0% to 0.3%), where microbiota were heavily dominated by *Firmicutes* and *Bacteroidetes* (Guard et al., 2015). Isaiah, Parambeth, Steiner, Lidbury, & Suchodolski (2017) recruited 18 healthy dogs to a study to compare the impact of exocrine pancreatic insufficiency on the fecal microbiome. *Proteobacteria* comprised 0.2% to 5.2% of the microbiota in this study, with a median abundance of 1.3%. Li, Lauber, Czarnecki‐Maulden, Pan, and Hannah (2017) examined lean and obese dogs fed high protein, low carbohydrate and low protein, high carbohydrate diets. Among the treatment groups, average *Proteobacteria* relative abundances in lean individuals ranged from 3.9% to 6.7%, with greater abundances in dogs fed the low protein diets (Li et al., 2017). In general, detailed information on diet composition from these studies was insufficient to dissect potential nutritional drivers of the diversity, but it is apparent that when fed a variety of commercial diets, the abundances of *Proteobacteria* in healthy animals varies considerably (Garcia‐Mazcorro et al., 2012; Hand et al., 2013; Handl et al., 2011).

### 4.2 Diet‐based studies in dogs

Diet‐related trends in humans have also extended to companion animals and interests in meat‐based, high animal protein and fat diets with minimal carbohydrates, and the use of prebiotics, have been gaining in popularity among pet owners in recent times. A number of studies have investigated the effects of such dietary trends on the dog fecal microbiome. These studies typically involve cohorts of research animals of similar breed, and detailed nutritional information of the diets used is available.

Raw meat diets in particular raise a number of food safety–related concerns, as the potential to carry pathogens such as *Escherichia coli*,* Salmonella,* and *Listeria*, and associated antimicrobial resistance determinants, is greater for the uncooked product (van Bree et al., 2018). Moreover, there are concerns around risks of infection to the pet owner (Schlesinger & Joffe, 2011), thus particular care around food hygiene and storage must be taken. As such, the impact of raw meat diets on the GI microbiome is of interest, and particularly with regard to *Proteobacteria*.

From studies to date, relative *Proteobacteria* abundances have varied considerably for raw meat feeding, which has often been contrasted to kibble diets (Bermingham et al., 2017; Kim, An, Kim, Lee, & Cho, 2017; Sandri, Dal Monego, Conte, Sgorlon, & Stefanon, 2017), although differences in study designs and diet formulations confound the ability to directly compare and interpret results. In the study by Sandri et al., a 70% raw beef skeletal muscle–based diet supplemented with carbohydrate fed to adult Boxer dogs resulted in significantly increased proportion (4.4%) of *Proteobacteria* in the raw meat fed animals compared to those fed a commercial extruded diet (1.3%) (Sandri et al., 2017). A highly significant shift in the abundance of *Enterobacteriaceae*, from 0.047% to 2.454% (*p *<* *0.01), and *Escherichia coli*/*Shigella* was seen, although all dogs were healthy (Sandri et al., 2017). In this study, both diets had similar protein concentrations. However, protein digestibility, and hence, the amount of protein entering the colon, was not measured (Sandri et al., 2017), which is a factor that influences microbiota composition. In contrast, in a trial that involved feeding Harrier Hounds a complete and balanced raw meat diet, *Proteobacteria* comprised an average of 0.56% (0.08%–1.8%) (Bermingham et al., 2017) after 9 weeks. This was compared to a commercially available kibble diet, where *Proteobacteria* comprised 1.27% (0.21%–2.01%), although the difference between raw and kibble‐fed animals was not significant (Bermingham et al., 2017). *Succinivibrio* and an unclassified member of the *Burkholderiales* were the only genera of *Proteobacteria* that displayed a significant difference in abundance between the two diets, with both being more abundant in kibble‐fed dogs (Bermingham et al., 2017). Similarly, in a study conducted in Korea (Kim et al., 2017), generally, lower abundances of *Proteobacteria* were observed in dogs fed a natural diet (>90% raw meat such as kangaroo, beef, and poultry; average 0.86%, range 0.27%–1.84%) than standard commercial kibble fed (8.67%, 0.05%–2.07%) (Students *T*‐test, *p *=* *0.07), where *Proteobacteria* were dominated by members of the *Enterobacteriaceae*. This study examined the fecal microbiomes of 11 mixed‐age, small breed dogs, recruited from a pet owner group based on their existing dietary regimes. Beloshapka and colleagues examined raw chicken‐ and raw beef‐based diets, both with and without inulin and yeast cell wall prebiotics, to beneficially alter the gut microbiota (Beloshapka et al., 2013). In general, the abundances of *Proteobacteria* averaged from 4.05% to 5.83% when fed each of the diet treatments, and these did not differ significantly with respect to either meat source, or addition of prebiotic. Few taxa significantly differed across treatments at the genus level. *Anaerobiospirillum* was more abundant when fed the chicken compared to the beef‐based diets. *Escherichia* abundances differed significantly, both in response to meat type, and prebiotic inclusion, across all diet groups, where they were present at 0.29%–1.69%.

High levels of cooked meat were examined in the study by Herstad and colleagues (Herstad et al., 2017). They observed a gradual decrease in *Proteobacteria* relative abundance from a kibble control diet through stepwise inclusions of cooked minced beef at 85% of the diet, where beef content strongly negatively correlated with average *Proteobacteria* abundance per treatment (Pearson's correlation, −0.979). However, in a comparison of *Proteobacteria* concentrations in the control diet (average 6.24% *Proteobacteria*, range 1.15%–14.34%) with the 85% beef treatment (3.98%, range 1.11%–11.85%), these differences were not highly significant (Students *T*‐test, *p *=* *0.10). *Sutterella* and *Anaerobiospirillum* dominated the most abundant *Proteobacteria* genera (Herstad et al., 2017).

A number of studies have investigated the impact of prebiotic and probiotic treatments (Beloshapka et al., 2013; Garcia‐Mazcorro, Barcenas‐Walls, Suchodolski, & Steiner, 2017; Garcia‐Mazcorro et al., 2011; Kerr, Forster, Dowd, Ryan, & Swanson, 2013; Middelbos et al., 2010; Panasevich et al., 2015), although again the impact of these on *Proteobacteria* levels and composition appears to be minimal. For example, the supplementation of a kibble diet with beet pulp dietary fiber, a common ingredient used in commercial dog food, provided a complex mixture of fermentable and nonfermentable carbohydrates (Middelbos et al., 2010). This resulted in significant increases in *Firmicutes* and decreases in *Fusobacteria*, but the relative abundances of *Proteobacteria* remained the same (Middelbos et al., 2010). A mixture of a fructooligosaccharides (FOS) and inulin was supplemented to dogs fed a range of diets, and led to significant shifts in the abundances of *Sutterella* among individuals. The responses across animals were highly variable, however, and may have depended on the baseline composition of the microbiota prior to prebiotic administration, and interaction with diet. Inclusion of potato fiber as a prebiotic source appeared to result in a small increase in the relative abundance of *Proteobacteria*, but this was not significant (Panasevich et al., 2015).

Taken together, the range of *Proteobacteria* abundances in clinically healthy dogs varies widely, and the influence of diet between studies is not clear cut. Certainly, harmonization of experimental and analytical methods would enable more direct comparisons between studies and overall trends to be deduced such as *via* a metastudy approach. Moreover, analyses of the microbiota metatranscriptomes would provide much greater insight into the metabolic activities of the *Proteobacteria* present and their functional contributions under different dietary regimes.

### 4.3 Proteobacteria in cats

*Proteobacteria* appeared to be generally less abundant in cats as compared to dogs, being detected at less than 4.3% in clinically healthy adult animals (Table 2), although considerably fewer studies were available to determine if this is a more general trend. However, in one study of the fecal microbiomes of cats with diarrhea (Suchodolski et al., 2015), among the 21 healthy subjects recruited to the control group, 0.26%–27.9% (median 4.79%) *Proteobacteria* was reported, and this appeared to be underpinned by few individuals that had up to 24% *Helicobacter*, or up to 7.4% *Campylobacter*. These results were not consistent with general observations from other studies (Table 2). These cats belonged to staff and students of a veterinary medical teaching hospital, so whether this aspect had contributed to a higher exposure of such microbial taxa remains unknown (Suchodolski et al., 2015).

In kittens, the relative abundance of *Proteobacteria* in the fecal microbiota generally appeared to be greater when they were fed diets with higher protein and fat content, than kibble, at around 3%–4% (Bermingham, Kittelmann, et al., 2013; Hooda, Vester Boler, Kerr, Dowd, & Swanson, 2013). In adult cats, *Proteobacteria* were detected at an average abundance of 1.1% when fed a wet (high protein, high fat) diet compared to 0.4% when fed a dry (moderate protein, low fat) commercial diet, with *Anaerobiospirillum* and *Sutterella* showing significantly higher abundances in the cats fed a cooked wet diet (Bermingham, Young, et al., 2013). In contrast, however, adults cats fed a raw meat‐based diet had only 0.4% *Proteobacteria* on average, compared to their kibble‐fed counterparts who averaged 2.4% (Butowski et al., unpubl.). Synbiotic administration did not appear to change the relative abundances of major phyla, although specific information on *Proteobacteria* abundance was not presented (Garcia‐Mazcorro et al., 2011), while FOS and inulin prebiotic treatments were reported to negatively impact *Proteobacteria* concentrations (Garcia‐Mazcorro et al., 2017).

### 4.4 Proteobacteria in the young

*Proteobacteria* are dominant members in the human neonatal gut, which is abundant in oxygen immediately post partum. The *Proteobacteria* are thought to play a key role in preparing the gut for colonization by the strict anaerobes required for healthy gut function by consuming oxygen, and lowering redox potential in the gut environment (Shin et al., 2015). Analyses of the GI microbiota of kittens from birth, and hence, an understanding of the dynamics of GI microbiome establishment in dogs and cats, have not yet been reported. Thus, the role of *Proteobacteria* at this critical stage of life in the dog and cat is yet to be confirmed. However, it would be reasonable to assume that they play similar roles in newborn dogs and cats, as in other mammals.

Two 16S rRNA gene studies examined the fecal microbiomes of newly weaned kittens (Bermingham, Kittelmann, et al., 2013; Hooda et al., 2013). In one study, *Proteobacteria* comprised, on average, 3% of the 16S rRNA sequences of kittens fed a high protein, low carbohydrate dry diet, compared to 1% or less when fed a moderate protein, moderate carbohydrate dry diet (Hooda et al., 2013). Fecal samples were taken at 8  (weaning), 12, and 16 weeks, where *Proteobacteria* abundances generally decreased over time (Hooda et al., 2013). In another study, the impact of maternal and postweaning diet was assessed in pre‐ and postweaned kittens at 8 and 17 weeks of age, respectively, using canned (high‐protein, high‐fat concentration) and kibbled (medium‐protein, medium‐fat concentration) diets (Bermingham, Kittelmann, et al., 2013). In this study, the relative abundance of *Proteobacteria* ranged from ~1% to 5% across the treatments, and although they appeared more abundant in canned‐fed kittens from kibbled‐fed mothers, this difference was not statistically significant. Moreover, *Proteobacteria* were generally more abundant at 8 weeks than at 17, but again, this was not significant. *Sutterella* was approximately three times more abundant in canned diet‐fed kittens from canned diet‐fed mothers than from kittens that had been exposed to the kibbled diet, either pre‐ or postweaning, and was the only member of the *Proteobacteria* whose abundance differed by postweaning diet (Bermingham, Kittelmann, et al., 2013). Shotgun metagenome studies have also examined the fecal microbiomes of kittens, where *Escherichia* and *Desulfovibrio* were among predominant bacteria, together with *Proteobacteria* in general, their relative abundances decreased as the kittens went from 8 to 16 weeks of age (Deusch et al., 2014).

## 5 PROTEOBACTERIA DIVERSITY IN THE DOG AND CAT FECAL MICROBIOME

In the dog, *Proteobacteria* was generally the fourth‐most abundant phylum behind *Firmicutes*,* Bacteroidetes,* and *Fusobacteria*, while in cats, it generally ranked behind *Firmicutes*,* Bacteroidetes,* and *Fusobacteria* or *Actinobacteria* (Tables 1 and 2). *Gammaproteobacteria* and *Betaproteobacteria* were most commonly observed, while other classes such as the *Alphaproteobacteria*,* Epsilonproteobacteria,* and *Deltaproteobacteria* were generally lower in abundance and not consistently detected. *Escherichia, Shigella, Succinivibrio*,* Anaerobiospirillum,* and *Sutterella* were among the most abundant recognized *Proteobacteria* genera reported in healthy individuals (Handl et al., 2011). *Escherichia* and *Salmonella* are members of the *Enterobacteriaceae* and while these genera are best known for their prominent pathogenic members, many isolates, even with diarrhea‐related virulence factors, are nonpathogenic and most likely contribute to normal microbiome function. Analysis of fecal samples from 70 diarrheic and 230 nondiarrheic domestic cats identified 15 enteropathogenic *E. coli* (EPEC) strains from 14 cats, of which only one cat was suffering from diarrheal symptoms (Morato et al., 2009). Additionally, dogs can be colonized by extended‐spectrum beta lactamase producing *E. coli* for extended periods of time (>6 months) without any clinical signs (Baede et al., 2015). A study in the United States to determine the prevalence of *Salmonella* in dogs and cats visiting veterinary clinics from 11 geographically dispersed veterinary testing laboratories indicated a low (<1%) overall prevalence in 542 cat fecal samples, and a prevalence of 2.5% (60 of 2422) in dogs, however, almost half the *Salmonella*‐positive animals were nondiarrheic (Reimschuessel et al., 2017). Similarly, the prevalence of *Campylobacter* spp. in rectal swab enrichments from 90 healthy dogs and 110 healthy cats was 36% and 16%, respectively (Bojanić et al., 2017).

*Succinivibrio* and *Anaerobiospirillum* are both succinate‐producing members of the family *Succinivibrionaceae* of the *Gammaproteobacteria,* and are recognized as part of the normal fecal microbiota of dogs and cats. *Succinivibrio* is commonly found in the rumens of cattle and sheep fed grain‐based diets, where they are involved in the digestion of starch and its breakdown products (Bryant & Small, 1956; Stackebrandt & Hespell, 2006), and it is presumed that they undertake similar roles in the GI microbiomes of dogs and cats. In contrast, *Anaerobiospirillum succiniciproducens* was first isolated from dog feces (Davis, Cleven, Brown, & Balish, 1976), and *A. thomasii,* a glucose, galactose, and maltose fermenter, was first isolated from the feces of healthy dogs and cats (Malnick, 1997). While *Anaerobiospirillum* is known to cause diarrhea and bacteremia in immunocompromised humans, dog ownership (and the potential for zoonotic transmission) is not recognized as an established as a risk factor for infection (Epstein, Ernst, Rogers, Carmody, & Aguero‐Rosenfeld, 2017a,b). *Sutterella* are members of the *Betaproteobacteria* (family *Alcaligenaceae*), where *Sutterella stercoricanis*, a Gram‐negative anaerobe, was first isolated from healthy canine faeces (Greetham et al., 2004). The asaccharolytic, nitrate‐reducing nature of *Sutterella* (Greetham et al., 2004) is suggestive of a key role in protein metabolism.

## 6 THE ROLES OF PROTEOBACTERIA IN DOG AND CAT GI MICROBIOME FUNCTION

An understanding of microbiota function, rather than its community structure alone, is necessary to truly recognize the contributions of the microbiota to host nutrition and wellbeing. Such knowledge will also contribute to developing strategies to improve host health. While the number of microbiome‐based studies in the dog and cat lag behind those of humans and related rodent models, insights into pet gastrointestinal microbiome functions are being pursued using similar molecular methods, revealing considerable similarities in function (Swanson et al., 2011), but also a number of distinct differences (Bermingham et al., 2017; Vázquez‐Baeza et al., 2016). A number of approaches to explore microbiome function from high‐throughput sequencing data have been used by the dog and cat microbiome research community, from inferring community functional gene composition from 16S rRNA gene profiles, to shotgun sequencing of metagenomic DNA. Notable by their current absence are studies that have examined the gene activities in microbiome samples. Metatranscriptomic studies, which sequence the total RNA within the sample, enable an in depth examination of the metabolically active members of the community and their specific contributions to GI metabolic, and other, processes. Although such studies will likely follow in due course, they will be more informative as a more diverse range of dog‐ and cat‐derived microbial isolates are brought into culture, characterized, and microbial reference genome information becomes available. These data will aid the interpretation of metagenomic and metatranscriptomic datasets, and allow more accurate predictions of “who is doing what”.

### 6.1 Microbiome function inferred from 16S rRNA gene profiles

In the more recent 16S rRNA gene‐based studies, the taxonomic composition of microbiomes has been extended to include predictions of microbiome function *via* the recruitment of available bacterial reference genome information that matches the 16S rRNA gene profiles (Garcia‐Mazcorro et al., 2017; Guard et al., 2015; Isaiah et al., 2017; Li et al., 2017) (Tables 1 and 2). Such analyses, most commonly implemented in the software, PICRUSt (Langille et al., 2013), have become more prevalent. However, the majority of available GI microbial reference genome data are from human gut (or model rodent) microbiome (Land et al., 2015; Turnbaugh et al., 2007) and rumen microbiome origin (Seshadri et al., 2018). Moreover, the *Proteobacteria* are such a diverse and heterogeneous group that 16S rRNA gene sequences are rarely sufficiently informative to identify specific strains. Current examination (16 Nov 2017) of the GOLD database of microbial genome sequencing projects (Mukherjee et al., 2017) retrieved 83 entries when the “Isolation host name” field was queried with “*Canis*”, and 37 entries for “*Felis*”. Almost all entries were from diseased hosts, with over a quarter of the dog and just under half of the cat entries being of viral origin. The remaining entries appear to be dominated by sequencing projects for pathogenic agents. Thus, it is not known how much the dog and cat commensal gastrointestinal microbial genomes differ to those from the currently available references available, and available reference data are likely to skew functional interpretations toward disease state microbiomes. The predictive accuracy of dog and cat microbiota function, using 16S rRNA gene‐based community data in conjunction with reference genome information, will only improve as more microbial genomes from healthy dog and cat sources become available. However, there is yet to be a systemic effort to sequence the genomes of microbes found in the dog and cat GI tract, and such information will be vital to identify unique functions of microbial taxa that are specific to the dog and cat hosts, and for accurate functional interpretation of metagenomic datasets generated from these sources.

### 6.2 Microbiome function from shotgun metagenome sequencing

Shotgun metagenome sequence datasets for dog and cat GI microbiota are now becoming more prevalent, and studies that are currently available in the literature are listed in Table 3. To analyze the microbiome to a similar depth as for 16S rRNA studies, the volume of shotgun sequence data required is several orders of magnitude greater, thus such studies are considerably more expensive than community profiling alone. However, advances in sequencing technologies and the decreasing costs of high‐throughput sequencing will likely see an increase in the number of shotgun metagenome sequencing studies for dog and cat GI microbiomes being undertaken in future. Shotgun sequencing not only provides information about the potential functions of the microbiota, but allows simultaneous capture of all major microbial groups (bacteria, archaea, fungi, and viruses), in contrast to amplicon‐based methods that have employed separate amplifications for each group (Kittelmann et al., 2013). Moreover, compared to marker‐dependent amplicon‐based procedures, there are fewer practical steps for potential biases to be incorporated (e.g., *via* primer‐based amplification biases) which influence data interpretation. However, the more complex bioinformatic pipelines required to analyze metagenomic sequence data may arguably induce more biases in silico. The presence of host DNA sequences, which can comprise a significant proportion of datasets, should be screened and removed before microbiota analysis is performed (Schmieder & Edwards, 2011).

**Table 3:** Healthy dog and cat fecal microbiota shotgun metagenome sequencing studies

Study description^a^	Subjects/age	Sequencing, analysis methods^b^, and accession numbers	Reference
General microbiome characterization			
Cats fed various commercial diets	Cat, 3–16 year, domestic long and shorthair (N = 5)	454 FLX Titanium (0.15 M reads); Galaxy, MG‐RAST, WebCARMA. NCBI SRA029158.2	(Tun et al., 2012)
Diet trial, treatments			
Dry control (30% CP, 19% fat, 1.4% fiber) vs. beet pulp containing diet (28% CP, 21% fat, 4.5% fiber)	Dog, ~20 month, mongrel and hound crosses, female (N = 6)	454 FLX Titanium (~0.5 M reads/sample); MG‐RAST. MG‐RAST 4444165 and 4444164; NCBI SRR054690	(Swanson et al., 2011)
Kibble diet with cellulose, FOS, or pectin	Cat, ~20 months, male (N = 4)	454 FLX Titanium (1.2–1.7 M reads/sample); MG‐RAST	(Barry et al., 2012)
Dry diets (HPLC and MPMC)	Kitten, sampled at 8, 12, and 16 weeks (N = 12)	Illumina, TruSeq DNAseq (~96 M reads/sample), MetaCV. ENA PRJEB4391	(Deusch et al., 2014)
Dry diets (HPLC and MPMC)	Kitten, sampled at 18, 30, and 42 weeks (N = 12)	Illumina, Nextera TruSeq SBS (~55 M reads/sample), MetaCV. ENA PRJEB9357	(Deusch et al., 2015)
Canned and kibbled diets	Kitten, 17 weeks (N = 20)	Illumina, TruSeq DNAseq (~4 M reads/sample), MG‐RAST. MG‐RAST 4629274.3–4629293.3	(Young et al., 2016)

a CP, crude protein; FOS, fructooligosaccharide; HPLC, high protein–low carbohydrate; MPMC, medium protein–medium carbohydrate diet.

b 454 refers to 454 GS FLX Titanium sequencing, Illumina refers to Illumina HiSeq2000. NCBI, ENA, and MG‐RAST database accession numbers provided where available.

A major bottleneck of metagenome‐based studies is the considerable computational requirements to analyze the volumes of data generated, and the biological interpretation of such analyses. Advances in computing power (Nobile, Cazzaniga, Tangherloni, & Besozzi, 2017) and the development of more efficient analyses methods (Buchfink, Xie, & Huson, 2015) have facilitated shotgun metagenome sequence analyses. Shotgun sequence data may be analyzed unassembled or assembled, where assembled data generate longer contiguous stretches of sequence that can more accurately retrieve hits to against reference databases. Indeed, in deeply sequenced datasets, ‘binning’ together assembled sequences of similar composition and abundance has the potential to recover full or near‐full genomes of highly abundant organisms (Parks et al., 2017; Tyson et al., 2004). However, assembly is likely to be poor for the vast majority of rare organisms (Sogin et al., 2006), and there is the potential to misassemble reads from closely related organisms.

MG‐RAST (Meyer et al., 2008) has been the main metagenome sequence annotation service used by researchers in the field of canine and feline GI microbiology (Barry et al., 2012; Swanson et al., 2011; Tun et al., 2012; Young et al., 2016). Sequences are assessed for quality and annotated for gene function and taxonomy against a number or reference databases. Annotated data can be viewed through the MG‐RAST web application, but more often is downloaded and explored using custom‐based analyses tailored to the focus of the project (Young et al., 2016). The first metagenomic studies of the dog and cat GI microbiome used 454 pyrosequencing technology, and generated from 150 K to 1.6 M reads per dataset (Barry et al., 2012; Swanson et al., 2011; Tun et al., 2012).

#### 6.2.1 Community composition biases revealed by metagenomic data

Shotgun sequence reads associated with rRNA gene sequences (or other marker genes of choice) can be identified from the dataset, and then used to generate a taxonomic profile of the microbial community using clustering techniques and comparison to referenced databases, similar to typical 16S rRNA gene analysis pipelines. From the taxonomic analysis of dog and cat GI shotgun metagenome data, it appears that the relative abundance of *Proteobacteria* is considerably greater than estimates from 16S rRNA gene amplicon‐based studies. For example, *Proteobacteria* represented 13%–15% of rRNA gene sequences *via* shotgun metagenome dataset analyses (Swanson et al., 2011), whereas only 5%–7% of sequences were classified as *Proteobacteria* in 16S rRNA gene V3 amplicon analysis of the same fecal samples (Middelbos et al., 2010). By comparison, *Bacteroidetes*/*Chlorobi* and *Firmicutes* each represented ~35% of taxa by metagenome analysis, but only comprised 27%–34% and 17%–24% of the amplicon‐based dataset, respectively. In contrast, the amplicon analysis appeared to overestimate *Fusobacteria* abundance (Swanson et al., 2011). In a separate study, *via* metagenome sequence analyses *Proteobacteria* comprised 8.7% of the community of kittens fed a canned diet (from mothers fed a kibbled diet) (Young et al., 2016), whereas 16S rRNA amplicon analyses of the samples placed the estimate for this group at ~4% (Bermingham, Kittelmann, et al., 2013). The *Firmicutes* also appear to be highly overrepresented by the 16S rRNA data compared to the metagenome sequence (Bermingham, Kittelmann, et al., 2013; Young et al., 2016). These discrepancies likely reflect biases resulting from the choice of amplification primers and conditions used to generate data (Soergel, Dey, Knight, & Brenner, 2012), as well as the different bioinformatics pipelines and reference databases used for each set of analyses. Thus, an additional level of caution is urged in the interpretation of data obtained *via* these various methods.

### 6.3 Proteobacteria contributions to microbioTA function

Metagenome studies have revealed primary functions that are abundant in the healthy dog and cat GI microbiomes, which include carbohydrate metabolism; protein metabolism; DNA metabolism, cofactors vitamins, prosthetic groups and pigments; amino acids and derivatives; cell wall and capsule; and virulence (Barry et al., 2012; Swanson et al., 2011; Tun et al., 2012; Young et al., 2016; Deusch et al., 2014, 2015).

**Figure 2:**
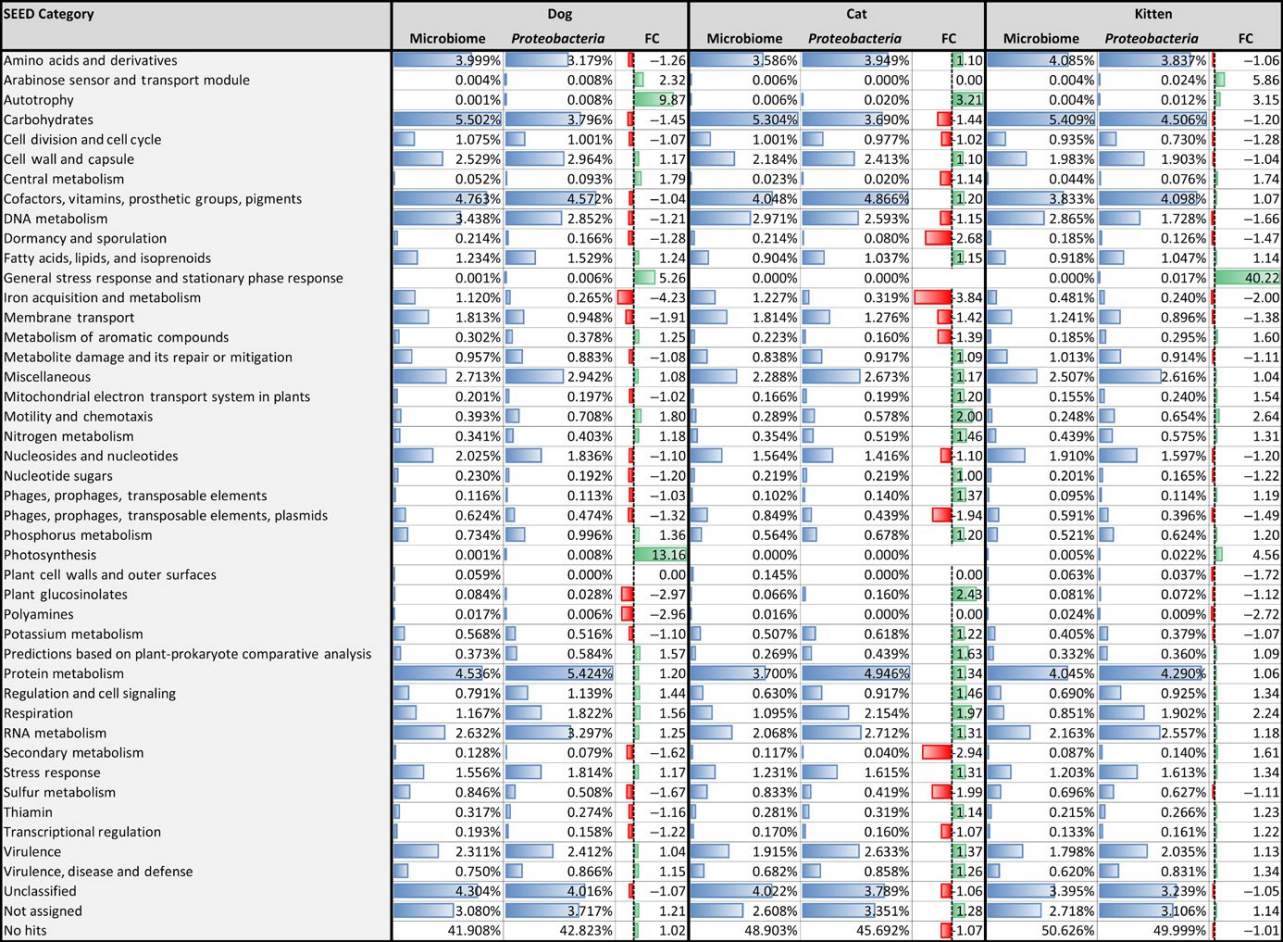
Functional profiles of representative healthy dog (Swanson et al., 2011), cat (Tun et al., 2012), and kitten (Young et al., 2016) fecal microbiota, compared to profiles from the subset of *Proteobacteria*‐associated reads within these datasets. Metagenomic shotgun sequence data were analyzed in MEGAN6 CE. The relative abundances of sequence hits for each SEED subsystem are shown as percentages with the length of the blue bar indicating their relative magnitude within the dataset, apart from the “No hits” category. Fold changes (FC) for *Proteobacteria* relative to microbiota abundances are shown, where fold increases are represented with green bars, and fold decreases (negative values) are represented by red bars.

The functional contributions that the *Proteobacteria* contribute to the microbiome cannot be readily deduced from the published data, as the reported functions for individual sequence reads are decoupled from taxonomic information (Barry et al., 2012; Deusch et al., 2014, 2015; Swanson et al., 2011; Tun et al., 2012; Young et al., 2016). We have therefore reanalyzed a representative metagenomic sequence dataset for each of the dog (Swanson et al., 2011), cat (Tun et al., 2012), and kitten (Young et al., 2016) fecal microbiota for which data were readily available (details of these datasets in Table 3) using MEGAN (Huson et al., 2016). MEGAN captures both functional and taxonomic information for each sequence read based on homology searches using DIAMOND (Buchfink et al., 2015) against the nr database. The resulting. daa files were meganized and visualized in MEGAN V6.10.2 Community Edition (Huson et al., 2016). This allowed direct relationships between the function and taxonomic origin to be examined, and thus, the functional roles of specific microbial taxa within complex communities, such as the *Proteobacteria*, to be explored. A comparison of the SEED subsystem profiles from each of representative dog, cat, and kitten pooled fecal microbiota, with those extracted from the reads that relate to *Proteobacteria* only, is presented in Figure 2.

At Level 1 of the SEED classification system, the most abundant *Proteobacteria* functions included “Protein Metabolism” (5.4%, 4.9%, and 4.3% of *Proteobacteria* reads in the dog, cat, and kitten datasets, respectively), “Cofactors, Vitamins, Prosthetic Groups, Pigments” (4.6%, 4.9%, and 4.1%), “Carbohydrates” (3.8%, 3.7%, and 4.5%), “RNA Metabolism” (3.3%, 2.7%, and 2.6%), and “Amino Acids and Derivatives” (3.2%, 3.9%, and 3.8%) (Figure 2). A considerable proportion of reads were to “Unclassified” sequences (4.0%, 3.8%, and 3.2%), “Not assigned” (3.7%, 3.4%, and 3.1%), or “No hits” (42.8%, 45.7%, and 50.0%) were observed (Figure 2), which reflect the extent to which gene and sequence identities are known for the pet gastrointestinal microbiota. The relative abundances of SEED subsystems for the whole community generally reflected those for the *Proteobacteria*. However, several classes appeared to have a considerably greater relative abundance in the *Proteobacteria* as compared to the whole microbiome: “Autotrophy” (3.2‐ to 9.8‐fold greater in *Proteobacteria*), which may reflect the metabolic versatility of the *Proteobacteria* as a whole and their ability to produce complex organic compounds from simpler substances; “General Stress Response and Stationary Phase Response” (ca. 40‐fold greater in the kitten, 5.2‐fold greater in the dog) and “Respiration” (1.6‐ to 2.2‐fold greater), which may reflect their respiratory abilities and ability to respond to stresses associated with aerobic respiration; and “Nitrogen Metabolism” (ca. 1.8‐ to 2.6‐fold greater) which is consistent with their contribution to protein metabolism (Figure 2). In contrast, the “Polyamines” (from undetected to ~3‐fold lower in *Proteobacteria*), “Iron Acquisition and Metabolism” (2‐ to 4.2‐fold lower), and “Carbohydrates” (1.2‐ to 1.5‐fold lower) are among SEED subsystems that appeared to be consistently underrepresented among the *Proteobacteria* in the datasets examined (Figure 2).

To better understand the specific functions that the *Proteobacteria* contributed to within their roles as protein degraders, sugar and oxygen utilizers within the gut, and their potential roles in pathogenicity, we examined the relative abundances of Level 2 SEED classification sequence hits for *Proteobacteria*, as compared to the whole microbiome. Due to the small number of datasets available, it was not possible to statistically determine the significance of apparent differences between datasets. As such we highlight subsystems that appeared to be consistently enriched for in both the dog and cat fecal datasets, or which had particularly large fold‐change differences.

#### 6.3.1 Proteobacteria contributions to protein metabolism

Among the “Protein Metabolism” Level 2 subsystems (Table S1), the “Putative TldE‐TldD proteolytic complex” subsystem had a 13‐fold greater relative abundance in *Proteobacteria* in the dog, and was 29‐fold greater in the cat dataset than the general microbiome. While best characterized in the capacity for biosynthesis of microcin B17, a peptide antibiotic (Ghilarov et al., 2017), *tldD* and *tldE* genes are highly conserved and common in prokaryotic genomes (Allali, Afif, Couturier, & van Melderen, 2002). They have recently been shown to encode a heterodimeric metalloprotease with unusual regulation of substrate specificity by directing unfolded polypeptides through a narrow channel with cleavage in a processive manner (Ghilarov et al., 2017). Rather than contributing to a general role in protein degradation, *tld* genes may contribute to important functions in maintaining protein quality control through the activation and degradation of specific products across a range of bacteria (Ghilarov et al., 2017).

“Peptide methionine sulfoxide reductase” was fivefold more abundant in the dog dataset, but was not among *Proteobacteria* sequences in the cat dataset. Moreover, “Periplasmic disulphide interchange” displayed 2.5‐fold greater abundance in the dog, and 2.1‐fold greater relative abundance in the cat datasets. These subsystems are associated with the repair of oxidatively damaged of proteins, where sulfur‐containing amino acids such as methionine and cysteine are particularly susceptible to damage by reactive oxygen species, which are normal byproducts of aerobic respiration. Methionine sulfoxide reductase reverses oxidative damage to methionine (Weissbach et al., 2002). Cysteine‐mediated disulfide bridges help maintain the tertiary structure of secreted proteins, and are introduced into proteins within an oxidative environment such as the periplasm of Gram‐negative bacteria (Lasica & Jagusztyn‐Krynicka, 2007). Hence, these functions are consistent to the role of *Proteobacteria* as facultative anaerobes, and are likely to contribute to normal protein function during aerobic respiration where additional pressures imposed by the generation of reactive oxygen species are higher.

Within the “Amino Acids and Derivatives” a number of subsystems were considerably more abundant in the *Proteobacteria* than in the general microbiome for both the dog and cat microbiome datasets (Table S2), including, “Putrescine utilization pathways” (13.2‐fold more abundant in the dog and 28.9‐fold more in the cat) and “Ketoisovalerate oxidoreductase” (3.8‐fold dog and 9.6‐fold cat). The putrescine utilization pathway (Puu pathway) gene cluster may have evolved to allow utilization of polyamines, which exist at relatively high concentrations in the gut, where it is found in *E. coli* and closely related enterobacteria, but is uncommon in other bacterial groups (Nemoto et al., 2012). The ketoisovalerate oxidoreductase catalytic domain (IPR019752; (Finn et al., 2017)) is generally involved in carbohydrate fermentation processes, but this subsystem was also likely also classified within the “Amino Acids and Derivatives” as the family includes pyruvate flavodoxin oxidoreductase, which in cyanobacterium, is required for growth on molecular oxygen when iron is limited (Bauer, Scappino, & Haselkorn, 1993). In addition, tyrosine and phenylalanine are both essential amino acids in the dog and cat (Council, 2006). The *Proteobacteria* may contribute to the supply of tyrosine and phenylalanine as the relative abundance of the “Tyrosine and phenylalanine metabolism in plants” subsystem in *Proteobacteria* was 2.1‐fold greater than the microbiome in general in the dog, and 1.8‐fold greater in the cat dataset. The relevance of the dog and cat fecal microbiome functions to those in plants is presumed to be due to general sequence similarities rather than specific plant functions, however.

#### 6.3.2 Proteobacteria contributions to carbohydrate metabolism

Within “Carbohydrate” (Table S3), there was little consistency among the top Level 2 classes that were overrepresented within the dog and cat microbiome. In common, the “Methylcitrate cycle” had *Proteobacteria* 12.5‐fold more highly represented than the general microbiota in the dog, and 22.4‐fold in the cat dataset. In *E. coli*, propionate, a major end‐product of gut fermentation, can be used as the sole source of carbon and energy using the methylcitrate cycle, to producing pyruvate, which can then be metabolized aerobically (Textor et al., 1997). Moreover, the dog dataset appeared to be highly enriched for the “CitAB” subsystem (13.1‐fold greater in *Proteobacteria* than the general microbiota) and a number of other Citrate Metabolism subsystems (Table S3). CitAB comprises a two‐component system involved in citrate fermentation (Scheu et al., 2012), although this subsystem was not detected in the cat dataset. Subsystems relating to the metabolism of a variety of carbohydrates and organic acids, such as d‐allose, d‐galactonate, “unknown carbohydrates”, and lactate utilization, were also more highly prevalent in the *Proteobacteria* in the dog microbiome dataset as compared to the cat. These differences may reflect differences in carbohydrate content of diets, where the dog study focused on beet pulp supplementation of a basal diet (Swanson et al., 2011), although the diets of the cats examined were not explicitly defined (Tun et al., 2012).

In the gut, mucins represent a major carbohydrate source for the microbiota (Pereira & Berry, 2017). Members such as *E. coli* colonize the mucus layer, and although they generally cannot degrade oligosaccharides or polysaccharides, they acquire mono‐ and disaccharides that result from extracellular hydrolysis of mucus and dietary polysaccharides by other microbial community members (Conway & Cohen, 2015). The ability of *E. coli* to colonize the mucus layer has been shown to depend on the ability to use a variety of mucin‐derived sugars, including gluconate, N‐acetylglucosamine, N‐acetylneuraminic acid, glucoronate, mannose, fucose, and ribose (Chang et al., 2004). However, the contributions of *Proteobacteria* relative to the general microbiota for subsystems related to the metabolism of these sugars did not show specific enrichment (Table S3). Thus, while mucins represent an important carbohydrate source in the gut, *Proteobacteria* do not appear to differ, more than the microbiota in general, in the level of their genetic potential to utilize these substrates.

#### 6.3.3 Proteobacteria contributions to aerobic respiration

Among the “Respiration” Level 2 subsystems (Table S4) a notably high proportion of categories were overrepresented in *Proteobacteria* relative to the whole microbiome for both dog and cat, with a number of subsystems associated with cytochrome C oxidase being among the most highly differentially abundant (~13‐ and 28‐fold in dog and cat microbiota, respectively). Cytochrome C oxidase is the last enzyme in the respiratory electron transport chain, and is critical for maintaining the electrochemical potential gradient across the cell membrane to facilitate ATP synthesis, while converting molecular oxygen into water (Ludwig, 1987). A number of general respiration subsystems related to the human gut microbiome were also relatively more abundant in the *Proteobacteria* (3.5‐ and 5.1‐fold, in dogs and cat, respectively), consistent with the *Proteobacteria* filling a role in aerobic respiration within the gut, and contributing to the redox homeostasis of the gut required for normal function of the strictly anaerobic microbial community members.

Subsystems involving formate dehydrogenases and formate hydrogenases were also more prevalent among the *Proteobacteria* as compared to the microbiome in general. In *E. coli*, the extracellular accumulation of formate at low pH induces biosynthesis of the formate hydrogen lyase complex, which enables formate transport into the cell and oxidation to CO_2_ and H_2_ (McDowall et al., 2014; Sawers, 1994). This process may generally contribute to the utilization of formate resulting from anaerobic fermentation by other gut microbes.

#### 6.3.4 Proteobacteria virulence

Within “Virulence” and “Virulence, Disease and Defence” (Table S5), the most prominent function that was enriched for in *Proteobacteria* was “Type 4 secretion and conjugative transfer” (7.5‐ and 18.4‐fold in the dog and cat, respectively), followed by “Mycobacterial MmpL2 membrane protein cluster” (4.1‐ and 5.8‐fold greater), “Mycobacterial MmpL6 membrane protein cluster” (1.7‐ and 2.9‐fold greater), and “Multidrug efflux pump in *Campylobacter jejuni* (CmeABC operon)” (2.4‐ and 1.4‐fold greater). Type 4 secretion systems are found in many bacterial species and represent significant functional diversity through conjugative transfer of genetic material, effector translocation, DNA exchange with the outside environment, biofilm formation, and lethal toxin delivery to bacterial neighbors (Grohmann, Christie, Waksman, & Backert, 2018). Short‐read polymorphisms associated with the CmeABC operon of *C. jejuni* have been associated with increased resistance to a number of quinolones and other antibiotics (Yang et al., 2017). *Proteobacteria* protein sequences with hits to Mycobacterial MmpL2 and MmpL6 membrane protein cluster SEED subsystems suggest that *Proteobacteria* are able to contribute related transporter functions to the microbiome. However, any potential role in virulence as for *Mycobacteria* (Domenech, Reed, & Barry, 2005), which are generally not detected in the healthy dog and cat, remains unclear. These proteins instead may simply be related to lipid transport in *Proteobacteria* taxa. Noteworthy, in the cat were the greater abundance of functions related to the resistance to heavy metals “Cadmium resistance” (9.6‐fold) “Copper homeostasis: copper tolerance” (2.6‐fold cat), although these were not as pronounced in the dog.

#### 6.3.5 Highly correlated taxa and gene functions involve *Proteobacteria*

**Figure 3:**
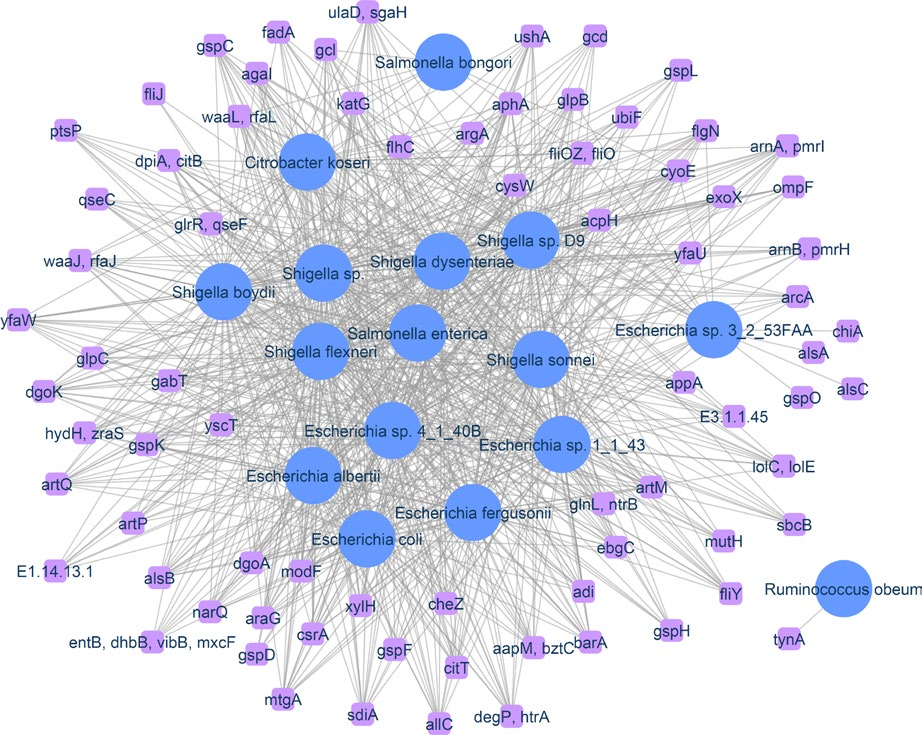
Network of the most highly correlated taxa (blue circles) and genes (purple squares) among the fecal microbiomes of 17‐week‐old kittens fed canned and kibbled diets. Kitten fecal microbiome sequence data were previously annotated in MG‐RAST (Meyer et al., 2008) for taxonomy and COG and KO functions as described (Young et al., 2016). Gene and taxon abundance network generated by sparse partial least squares regression using the spls function in the mixOmics package (Lê Cao & González, 2009) for R, using a correlation cut off of > |0.9|. The networks were viewed in Cytoscape 3.5.1 (Shannon et al., 2003)

Correlation analyses between microbial taxa and gene abundances can be undertaken using annotated shotgun sequencing data to explore functions that are potentially uniquely contributed to by specific taxa. The kitten fecal metagenome study (Young et al., 2016) is comprised of 20 shotgun sequence datasets, which has allowed correlation analyses to be performed (Figure 3). Interestingly, the highest correlations observed (*R* ≥ 0.9) involved many members of the *Proteobacteria* including *Escherichia, Salmonella,* and *Shigella,* which correlated with a wide variety of genes predominantly involved in amino acid, sugar, and sulfate transport systems, secretion systems, and lipopolysaccharide biosynthesis (Figure 3). These genes are in keeping with known functions of these taxa, but whether or not they are from these taxa would require further validation. Given the tight network of genes with these closely related taxa, there is good likelihood that this was the case.

*Ruminococcus* also featured in this analysis, but its abundance only highly correlated with one gene, *tynA,* which encodes a primary amine oxidase.

## 7 SUMMARY AND FUTURE DIRECTIONS

In the fecal microbiomes of healthy dogs and cats, *Proteobacteria* were detected in almost all individuals examined, where they were generally the third‐ to fifth‐most abundant bacterial phylum present. *Proteobacteria* abundances and diversity were variable, however, and 16S rRNA gene‐based surveys showed that the gene copy abundance ranged widely in dogs, from 0% to ~22%, while in cats, average abundances were generally <5%. Commonly observed genera include *Escherichia*,* Shigella*,* Succinivibrio*,* Anaerobiospirillum*, and *Sutterella*, although the relative abundances of each varied between individuals and studies. It is difficult to discern the key factors that influence *Proteobacteria* abundance and diversity, given the confounding factors between studies, however, that diet is a key driver of microbiota composition is clearly demonstrated within controlled studies. Metagenomic sequence data show that the *Proteobacteria* encode a variety of functions, which most prominently include protein and amino acid metabolism, carbohydrate metabolism, and cofactor/vitamin/prosthetic group/pigment metabolism. Compared to the fecal microbiota in general, the *Proteobacteria* appear to confer more abundantly to a number of functions that relate to their ability to grow aerobically such as respiration, and help maintain energy efficiency and integrity in the high redox potential environment generated while doing so, such as the utilization of propionate as a carbon source, and repair of protein from oxidative damage. These insights have been obtained from datasets based on relative gene abundances, and using reference genome and sequence data whose relevance to the dog and cat GI microbiota remains unclear. However, the functional contributions of *Proteobacteria* to microbiome function will become clearer with (1) greater efforts to cultivate and characterize diverse microbial representatives from dog and cat GI sources, (2) greater availability of reference genomes from dog‐ and cat‐derived strains, (3) greater depth of metagenomic sequencing, given the lower representation of *Proteobacteria* within the microbiome, and (4) metatranscriptome studies to identify metabolically active members of the microbiome and the pathways they utilize. Such information will allow us to further determine the roles and contributions of *Proteobacteria* to microbiome function in healthy dogs and cats, including their interactions with other members of the microbiota, as well as during dysbiosis and disease. As in other mammalian hosts, these observations point to the *Proteobacteria* occupying a unique ecological niche in the microbiome, where they broadly contribute to protein and carbohydrate metabolism, and also maintaining oxygen homeostasis in the gastrointestinal tract of the healthy dog and cat.

## CONFLICT OF INTEREST

None declared.
